# Frailty as a Predictor of Adverse Outcomes among Spanish Community-Dwelling Older Adults

**DOI:** 10.3390/ijerph191912756

**Published:** 2022-10-05

**Authors:** Ascensión Doñate-Martínez, Tamara Alhambra-Borrás, Estrella Durá-Ferrandis

**Affiliations:** Polibienestar Research Institute, University of Valencia, 46022 Valencia, Spain

**Keywords:** frailty, predictive performance, disability, falls, quality of life, use of healthcare resources

## Abstract

Spain is one of the European countries with the oldest populations. The prevalence of frailty among Spanish older people ranges from 8.4 to 29.4% and currently, is one of the most relevant public health challenges. The Tilburg Frailty indicator (TFI) has been widely used in the community and in healthcare settings for assessing frailty. The objective of this study is to evaluate the predictive performance of the TFI for several adverse outcomes among Spanish community-dwelling older adults. The predictive performance was tested through linear regression analyses and receiver operating characteristics (ROC) curves. A total of 552 Spanish older adults composed the study sample. Participants were assessed at baseline and after 6 months. Main results showed that frailty was strongly and significantly correlated with disability, physical health, mental health and falls efficacy. The TFI score predicted most of these adverse outcomes. The ROC analyses confirmed the acceptable predictive performance of the total frailty. This study provides new evidence confirming that the TFI is a valid tool to predict several adverse outcomes in Spanish older adults, which may allow professionals to plan and activate health and social care resources to support frail patients’ needs.

## 1. Introduction

Frailty is one of the most relevant public health challenges to be faced as a consequence of population ageing. A recent systematic review and meta-analysis studied the prevalence of frailty in 62 countries [1]. According to this study, it is estimated that 17 per cent of older adults are frail, and 45 per cent are pre-frail. Paying special attention to Europe’s figures, another recent literature review and meta-analysis performed with data from 22 European countries showed that the prevalence of frailty in community settings was 12 per cent and 45 per cent in non-community settings [2]. Moreover, the results of two large population-based longitudinal studies, the Survey of Health and Retirement in Europe (SHARE) and the Study on Global AGEing and Adult Health (SAGE), found the highest prevalence of frailty in Spain, Italy and Poland [3]. In particular, O’Caoimh et al. (2018) [2] reported that the prevalence of frailty among Spanish people aged 65 years and over living independently in the community ranged from 8.4 to 29.4 per cent. A similar rate was found in a later study in which the frailty prevalence was estimated at 26.2 per cent among community older adults [4]. In addition, the meta-analysis by O’Caoimh et al. (2018) [2] found that Spain was one of the most active European countries publishing studies reporting data on frailty prevalence rates; showing that frailty it is a topic of interest within the Spanish context.

It is important to highlight that physical frailty is associated with increased risk of death and multimorbidity [5] as well as polypharmacy [6]. In addition, frail older adults are at higher risk of experiencing recurrent falls in comparison to robust older people [7]. Furthermore, quality of life (QoL) is also affected by frailty, showing an inverse association between frailty, pre-frailty and QoL among community-dwelling older adults [8]. In this regard, frailty was also associated with poor QoL among specific groups of older patients, such as those with cancer [9], chronic obstructive pulmonary disease [10] or heart failure [11]. Additionally, frail older adults are at a higher risk of hospitalization than robust older adults [12]. Finally, yet importantly, the limitations in performing daily activities or the dependency status that frail adults may experience are also a risk factor to be isolated from others and feeling lonely [13].

It is evident that frailty affects different dimensions of a person’s health status. Nevertheless, frailty has been traditionally considered from a clinical and medicalized approach. In line with this approach, in 2018, the frailty definition was introduced to the National Library of Medicine’s MeSH database as “a state of increased vulnerability to stressors, following declines in function and reserves across multiple physiologic systems, characterized by muscle weakness, fatigue, slowed motor performance, low physical activity and unintentional weight loss”. Additionally, the conceptualizations most commonly studied in the literature, and also used in clinical practice, are based on physical frailty [14]. These are the Frailty Phenotype [15] and Frailty Index [16,17]. In 2001, Fried et al. (2001) [15] proposed a physiologically-based definition of frailty in which community-dwelling older persons are considered frail if they have three or more of the following five criteria: unintentional weight loss, self-reported fatigue/exhaustion, weak handgrip strength, slow walking speed and low level of physical activity. In regard to the Frailty Index (FI), Mitnitski et al. (2001) [16] and Rockwood et al. (2005) [17] operationalized frailty as an index by counting various clinical deficits over time, such as the presence and severity of current diseases, functional impairments and laboratory abnormalities, among others.

These physical-based definitions are not immune from criticism. Several authors have considered that this biomedical approach to frailty lacks a holistic consideration that looks at the whole person, which can lead to fragmented care [18,19]. To overcome this narrow approach, Gobbens et al. (2010a) [20] developed an integral and multidimensional conceptual definition of frailty, based on a literature review and a posterior consultation with 20 multidisciplinary and international experts, in which frailty is: “a dynamic state affecting an individual who experiences losses in one or more domains of human functioning (physical, psychological, social), which is caused by the influence of a range of variables and which increases the risk of adverse outcomes”. This comprehensive approach to frailty includes physical, psychological and social domains, which were defined by Gobbens et al. (2010a) [20] as follows: physical frailty consists of unexplained weight loss, physical unhealthy, difficulty in walking, difficulty in maintaining balance, vision problems, hearing problems, lack of strength in the hands and physical tiredness; the psychological domain of frailty includes problems with memory, feeling down, feeling anxious or nervous and unable to cope with problems; finally, the social domain comprises living alone, lack of social relations and lack of social support.

Despite the efforts to reach a consensus on the definition of frailty [21], there is still an ongoing discussion on a common approach for addressing frailty. Thus, it is important to use tools and instruments that evaluate this condition from a comprehensive approach that can guide professionals to take appropriate interventions on multiple life domains. To date, there are several instruments aimed to assess multidimensional frailty, such as the Edmonton Frail Scale (EFS) [22], the Groningen Frailty Indicator (GFI) [23], Kihon Checklist (KCL) [24] or the Tilburg Frailty Indicator (TFI) [25]. Among them, the TFI has been widely used in the community as well as in different healthcare settings—i.e., hospitals or emergency departments—and it has strong evidence of its reliability and validity [26]. Given that, the TFI is very easy to administer and score, it is a cost-efficient tool to study the prevalence of frailty in large-scale samples. Regarding, the predictive performance of TFI has been analyzed in several studies. Among those, TFI has been proved as a valid instrument to predict the following adverse outcomes: disability and health care utilization [27] and falls [28] in Dutch community-dwelling elders; disability and QoL in a sample of community-dwelling elderly individuals from Portugal [29]; and hospitalization, falls and loss in functional capacity in activities of daily living (ADL) in Brazilian older users of primary health care services [30]. However, more research is needed to support evidence of the predictive performance of TFI to predict adverse outcomes among older populations in other countries and in other care settings. This is key, not only for the older populations but also for the healthcare system, as the TFI is among those few instruments assessing frailty from a comprehensive approach (including physical, psychological and social domains of frailty).

Previous studies with Spanish older people have showed the relationship between frailty and adverse outcomes using physical-based instruments to measure frailty, such as the physical indicators proposed for SHARE [31] and the Fried phenotype [32]. Nevertheless, to the best of the authors’ knowledge, to date, there are no studies providing answers to this research question: is the TFI a valid tool to predict adverse outcomes beyond frailty among Spanish older adults? Therefore, the objective of this study is to evaluate the predictive performance of the TFI for several adverse outcomes (disability, fall risk, quality of life and use of healthcare resources) in a sample of Spanish older people. This target population is particularly important as Spain is one of the European countries with the oldest populations [33].

## 2. Materials and Methods

### 2.1. Design

This is a cross-sectional and longitudinal cohort study design using data from two research projects funded by the European Commission Health Programme. On the one hand, the Urban Health Centers Europe (UHCE) project, a longitudinal study aimed to develop and test a coordinated preventive care approach for healthy ageing in five European countries. On the other hand, the APPropriate CARE paths for frail elderly patients (APPCARE) project, a longitudinal study aimed to analyze the effectiveness of comprehensive geriatric assessment models to reduce hospital admissions among older adults in three European Countries. These two projects were selected because both conducted pilot studies in Spain (in particular, Valencia region), with the same target population (>70 community-dwelling older adults), within the same time frame, and both projects used the TFI to measure frailty among participants. Both studies designs have been described in detail elsewhere [34,35]. In the present study, only data of Spanish participants, in particular from the Valencia region, from both projects were used.

### 2.2. Study Population and Data Collection

From these two projects, a total sample of 723 Spanish older adults was recruited (500 from UHCE project and 223 from APPCARE project). The study population consisted of community-dwelling people aged 70 years or older from the city of Valencia (Spain). Among the exclusion criteria, participants were not eligible to participate if they were not able to comprehend the information provided in the local language or if they were not able to make an informed decision regarding their participation in the study.

From the total sample, 552 older adults completed both baseline and follow-up self-reported questionnaires (response rate of 76.3 per cent). The follow-up assessment was performed within six months of the prior assessment.

All participants included in this study provided written informed consent. Ethical committee procedures were followed in both research projects, and approval was provided by the Ethics Committee of Consorcio Hospital General Universitario de Valencia (UHCE project; ref. 29/01/2015; CICHGUV-2015-01-29) and the Ethics Committee of Hospital Universitario y Politécnico La Fe (Valencia, Spain) (APPCARE project; ref. 2016/0500).

### 2.3. Measures

#### 2.3.1. Socio-Demographic Variables

Socio-demographic characteristics of interest were age, sex, marital status, living situation and educational level. See Table 1 for the response categories.

#### 2.3.2. Frailty

Frailty was measured with the TFI [25], a 15-item instrument that includes questions on physical, psychological and social domains of frailty. The total TFI score range from zero to 15 with higher scores indicating higher levels of frailty, and a person is considered frail if the total score is five or higher. The TFI showed moderate internal consistency (Cronbach’s alpha = 0.712).

Regarding the TFI subscales, the physical domain contains eight items (scores ranging from 0 to 8) on physical health, weight loss, walking, balance, hearing, vision, strengths in hands and tiredness. The psychological domain is assessed through four items (scores ranging from 0 to 4) on memory, feeling down, anxiety or nervousness and coping with problems; and the social domain comprises three items (scores ranging from 0 to 3) on living alone, missing people and receiving enough support.

#### 2.3.3. Adverse Outcomes

Disability was measured using the Groningen Activity Restriction Scale (GARS) [36]. This 18-item scale measures the level of independence and its scores range from 18 to 72, with higher scores indicating lower levels of independence. GARS showed an excellent internal consistency (Cronbach’s alpha = 0.975).

Fall risk was measured by four indicators: two items on falls and the number of falls; How many times did you fall in the past 12 months?), an item on fear of falling (Are you afraid of falling? Yes/No), and by the Falls Efficacy Scale International (FES-I) [37], the 7-item short version. FES-I scores range from 7 to 28, and higher scores indicate higher concern about falling. The internal consistency of FES-I was excellent (Cronbach’s alpha = 0.929).

Quality of life was assessed using two different measures, the 12-item short form health survey version II (SF-12v2) [38] and the Jong–Gierveld loneliness scale [39]. The SF-12v2 consists of two subscales, the physical and mental health components, and in both subscales, scores range from 0 to 100, with higher scores indicating higher levels of quality of life. The 6-items Jong–Gierveld loneliness scale scores range from 0 to 6 with higher scores representing higher levels of loneliness. Validity evaluations showed adequate internal consistency of both measures (SF-12v2, Cronbach’s alpha = 0.917, and Jong–Gierveld loneliness scale, Cronbach’s alpha = 0.693).

Finally, four indicators of the use healthcare resources were included as adverse outcomes. On the one hand, indicators on the healthcare utilization, as the number of visits to general practitioner (GP) (In the past 12 months, how many times have you visited a GP for yourself?) and the number of hospital admissions (In the past 12 months, how many times have you been admitted to a hospital?). On the other hand, indicators on the use of medications, as polypharmacy (Do you currently take five or more different medicines? Yes/No) and presenting difficulties in taking medications (Is it difficult for you to take your medicines as prescribed? Yes/No).

### 2.4. Statistical Analysis

Data were analyzed using IBM SPSS Statistics 26. In all the analyses conducted, a *p*-value < 0.05 was considered statistically significant.

Pearson’s correlations between the three frailty domains and the adverse outcomes were performed using both baseline and follow-up measures. Correlations values from 0.1 to 0.3 were considered to be small, from 0.3 to 0.5, moderate, and from 0.5 to 1.0, strong [40,41].

Then, the predictive performance of the TFI was examined through linear regression analyses and receiver operating characteristic (ROC) curves.

First, linear regression analyses were conducted to examine the effect of frailty–physical, psychological and social frailty domains (independent variable) on adverse outcomes—disability, physical health, mental health, loneliness, falls efficacy, number of falls, visits to GP, and hospital admissions (continuous dependent variables); and logistic regression analyses were performed to study the effects of the three frailty domains (independent variable) on the other adverse outcomes—falls, fear of falling, polypharmacy and difficulty in taking medications (categorical dependent variables).

These linear and logistic regression analyses were conducted, using follow-up measures, to explore the cross-sectional associations between the frailty domains and the adverse outcomes. In addition, the longitudinal associations among these variables were also analyzed using linear and logistic regression analyses. For the longitudinal analyses, the baseline measures of the frailty domains and socio-demographic characteristics and the follow-up measures of the adverse outcome variables were used.

All regression analyses were conducted in two sequential steps. The first step included the socio-demographic characteristics—age, marital status, living situation and educational level. In the next step, the frailty domains were added to the model. The interest of this regression model is whether the model from step two (frailty domains), after controlling for the socio-demographic characteristics, explained the adverse outcomes better than the model from step one (socio-demographic characteristics).

Finally, ROC curves were used to evaluate the predictive performance of frailty using the TFI. The ROC analyses were applied to the following adverse outcomes: disability, physical health, mental health, loneliness, falls efficacy, falls, fear of falling, polypharmacy and difficulty in taking medications.

All these analyses have been performed in line with those carried out by other authors studying the predictive performance of the TFI [28].

The ability of the TFI to predict each of these adverse outcomes was assessed using the area under the curve (AUC), for which values > 0.7 were considered acceptable and >0.9 outstanding [42]. In addition, sensitivity and specificity were estimated for each adverse outcome at each TFI cut-off point.

## 3. Results

### 3.1. Socio-Demographic Information of the Sample

In regard to the socio-demographic characteristics, participants had a median age of 79.1 and more than 60 per cent were female. Regarding frailty, at baseline the average score on total frailty was 5.6 and 5.4 at follow-up. These scores indicate that participants were frail, according to the TFI cut-off point 5. Table 1 presents a complete overview of the participants’ characteristics, including the adverse outcomes.

### 3.2. Correlations between the Study Variables

Pearson’s correlations between the study variables—total frailty (TFI total score), frailty domains and adverse outcome variables—were conducted. Table 2 shows the correlations between the study variables at both baseline and follow-up.

At baseline, significant correlations were found among all the study variables (*p* ≤ 0.01), with the exception of the social frailty domain and the number of visits to the GP that were not significantly related. Moreover, at follow-up, significant correlations were found among all the study variables (*p* ≤ 0.05), with the exception of the psychological and social frailty domains that were not significantly related to hospital admissions; and the social frailty domain that, as happened at baseline, was not related the number of visits to the GP.

Significant correlations (*p* ≤ 0.01) between total frailty and the frailty domains were considered strong (0.5 to 1.0), and the correlations among the frailty domains were also found significant (*p* ≤ 0.01) but moderated (0.3 to 0.5).

For our Spanish sample, correlations between total frailty and most of the adverse outcomes ranged from moderate (0.3 to 0.5) to strong (0.5 to 1.0) at both baseline and follow-up. Especially, strong correlations were found among total frailty and disability (0.607 at baseline and 0.637 at follow-up), physical health (0.706 at baseline and 0.694 at follow-up), mental health (0.608 at baseline and 0.703 at follow-up) and falls efficacy (0.590 at baseline and 0.618 at follow-up). On the contrary, small but significant correlations were found between total frailty and the number of visits to GP (0.167 at baseline and 0.188 at follow-up) and hospital admissions (0.256 at baseline and 0.104 at follow-up).

### 3.3. Predictive Performance: Regression Analysis

#### 3.3.1. Cross-Sectional Regression Analyses

Cross-sectional effects of the frailty domains on adverse outcomes were calculated using regression analyses. In particular, linear regression analyses were performed for the continuous adverse outcomes variables and logistic regression analyses for the categorical adverse outcomes variables.

Table 3 shows the cross-sectional effects of frailty on the continuous adverse outcomes after controlling for socio-demographic characteristics. All three frailty domains together significantly explained (*p* < 0.001) the following adverse outcomes of our Spanish sample: disability, physical health, mental health and falls efficacy. The explained variance (R2) for these variables was: 37.8 per cent, 51.4 per cent, 50.4 per cent and 36.2 per cent, respectively. Besides, 22.7 per cent of loneliness scores were significantly explained by psychological and social frailty domains (*p* < 0.001), and 9 per cent of the number of falls was explained by physical frailty (*p* < 0.001). Among our population, physical frailty was the only frailty domain that had a significant effect on visits to GP, and hospital admissions were not explained by any of the frailty domains.

The logistic regression analyses presented in Table 4 showed that, after controlling for socio-demographic characteristics, the frailty domains together significantly explained (*p* < 0.001) the adverse outcome of difficulty in taking medications. In particular, 52.8 per cent of the variance of this adverse outcome. Physical frailty was the frailty domain that had a significant effect (*p* < 0.001) on all the adverse outcomes analyzed. Social frailty significantly explained having suffered a fall (falls), fear of falling and difficulty in taking medications with *p*-values of 0.022, 0.045 and 0.019, respectively; and as previously said, psychological frailty only explained difficulty in taking medications (*p* ≤ 0.005). For the full model, including socio-demographic characteristics, significant regression equations were found for all adverse outcomes, with the exception of hospital admissions (see the last row of Table 3 and Table 4).

#### 3.3.2. Longitudinal Regression Analyses

Longitudinal effects of the frailty domains on adverse outcomes were also calculated through linear regression analyses for the continuous variables and using logistic regression analyses for the categorical variables.

Table 5 presents the effects of frailty on the adverse outcomes after 6 months, and after controlling for the socio-demographic characteristics of the Spanish participants. Frailty domains significantly explained (*p* < 0.001) the following percentage of variance for each of these adverse outcomes: disability (34.9 per cent), physical health (38.6 per cent), mental health (37.4 per cent), falls efficacy (30.9 per cent), loneliness (14.3 per cent). The adverse outcomes: visits to GP (*p* ≤ 0.000), hospital admissions (*p* ≤ 0.016) and the number of falls (*p* ≤ 0.000) were only explained by the physical frailty domain.

Longitudinal analyses for the categorical variables presented in Table 6 showed that the resulting model of the three frailty domains, after controlling for the socio-demographic characteristics, significantly explained (*p* ≤ 0.000) having suffered a fall (falls), fear of falling, polypharmacy and difficulty in taking medications. Physical frailty was again the frailty domain that significantly predicted (*p* ≤ 0.001) all the adverse outcomes. Psychological frailty predicted falls (*p* ≤ 0.041) and difficulties in taking the medication (*p* ≤ 0.007). Finally, social frailty was not responsible for any of these adverse outcomes.

Again, significant regression equations were found for the full model for all adverse outcomes (see the last row of Table 5 and Table 6).

#### 3.3.3. Predictive Performance: ROC Curves

ROC curves were plotted to prove the cross-sectional and longitudinal predictive performance of total frailty, measured through the TFI, for predicting the following adverse outcomes: disability, physical health, mental health, loneliness, falls efficacy, falls, fear of falling, polypharmacy and difficulty in taking medications.

In regard to cross-sectional predictive performance of TFI, the resulting Area Under the Curve (AUC) with respect to disability, physical and mental health, and falls efficacy, fear of falling, polypharmacy, difficulty in taking medications was >0.7, which is considered acceptable. The AUCs for loneliness and falls were mediocre (0.694 and 0.668, respectively). The longitudinal predictive performance of frailty showed similar results as the cross-sectional analyses, determining that the resulting AUCs were acceptable (>0.7) for disability, physical and mental health, falls efficacy, fear of falling, polypharmacy and difficulty in taking medications; and mediocre (AUC between 0.6 and 0.7) for loneliness and falls.

Contingency tables were used to calculate sensitivity and specificity for the TFI. In most cases, using cut-off point of 5 and 6 for total frailty showed the best discriminative ability, as shown in the Supplementary Material.

## 4. Discussion

The aim of this study was to determine the predictive performance of the TFI, analyzing data cross-sectionally and longitudinally from a sample of Spanish older adults. The study results confirmed the predictive performance of the TFI to predict adverse outcomes among Spanish older populations, showing the relevance of using the TFI and its comprehensive approach of frailty within the healthcare setting and in other care settings.

Main results showed that total frailty was strongly and significantly correlated with several adverse outcomes: disability, physical health, mental health and falls efficacy. These results are in line with the profile of Spanish older adults with frailty, who use to experience a poorer self-perceived health status [4,43], higher degrees of disability [44,45], higher use of polypharmacy [46] or higher use of health resources [47]. More specifically, physical frailty was the TFI domain more strongly correlated to these adverse outcomes both at baseline and follow-up and to hospital admissions at follow-up. However, the psychological frailty domain of our Spanish sample was only associated with mental health. Finally, the social frailty domain did not show any strong correlations with adverse outcomes; only loneliness was found to be moderately correlated to the social frailty domain of the TFI.

Regression analyses showed that the total TFI score predicted the following adverse outcomes: disability, physical health, mental health, falls efficacy and difficulty in taking medications, in both cross-sectional and longitudinal analyses. In addition, the three frailty domains together predicted loneliness, having suffered a fall, fear of falling and polypharmacy, in only longitudinal analyses.

In other longitudinal studies, moderate and strong associations were also found between total frailty measured with TFI and adverse outcomes—such as disability, health care utilization and quality of life—one or two years later [48]. In addition, TFI showed satisfactory predictive ability for decreased quality of life, falls or disability in previous studies [30,49,50].

On the one hand, physical frailty was the TFI domain with a more significant effect on all the adverse outcomes analyzed, as noted before by other authors [27,28,29]. Additionally, the physical domain was the only one explaining visits to healthcare resources such as to GPs (cross-sectional) and hospital admissions (longitudinal). These findings are also in line with the results of the study conducted by Santiago et al. (2018) [30] showing that physical frailty had a significant effect on hospitalizations. On the other hand, psychological frailty explained the difficulty in taking medications both at baseline and follow-up, as well as loneliness (cross-sectional) and falls (longitudinal). These results can be supported by the evidence from other authors stating that poorer self-reported mental health can be associated with lower levels of medication adherence because of the decline at the cognitive level [51], more feelings of loneliness as a consequence of symptoms of depression, such as anhedonia, decreased vitality or low self-esteem [52] or a higher risk of falls that can be related to recurrent changes in cognition, behavior and mental state [53]. Moreover, our results showed that social frailty was associated with loneliness, falls, fear of falling and difficulty in taking medications in cross-sectional analyses, but for none of the adverse outcomes longitudinally. Similar results have been reported in other longitudinal studies in which no predictive association between the social domain of the TFI and these adverse outcomes was found [21,29,48].

Additionally, the resulting AUC of the ROC curve analyses conducted in this study confirmed the acceptable predictive performance of the total frailty for disability, physical health, mental health, falls efficacy, fear of falling, polypharmacy and difficulty taking medications (values between 0.723 and 0.875). AUC values for disability (>0.80) were similar to those obtained by other authors in the Netherlands using also the GARS score [28,48]. Lower AUC values were found in other studies performed again in the Netherlands (0.66) using the GARS [50], in Portugal (0.63–0.72) using the Barthel Index and the Lawton and Brody Scale [29] and in China (0.68–0.73) measuring disability using the Katz Index [54]. In our study, total frailty showed mediocre predictive performance for loneliness and falls (AUCs between 0.659 and 0.694); similar AUC values for falls prediction were found in Gobbens et al. (2020) [28] (0.663–0.706). Nevertheless, a recent cohort study [55] found a lower predictive performance (AUC of 0.59) of the TFI predicting falls. We did not find data in the literature to compare AUC results regarding quality of life (physical and mental), loneliness, fear of falling, polypharmacy or difficulty talking medications. However, Dong et al. (2017) [54] studied the predictive performance of TFI also on depression and low social support, which AUC values (0.83 and 0.77 respectively) were similar to our values for mental health (0.809–0.869) and loneliness (0.692–0.694).

The most optimal cut-off points for total frailty in Spanish community-dwelling older adults were 5 and 6. On the one hand, sensitivity values were better for all adverse outcomes with the cut-off point of 5 assessed cross-sectionally and longitudinally. However, the cut-off point of 6 performed with higher specificity values in all outcomes and in both analyses. These results are in line with Gobbens et al. (2010b) [25], who established the original TFI cut-off point for frailty at ≥5 points. Other studies about the predictive validity of TFI showed optimal cut-off points of 6 in Portugal [29] or 4 in China [54].

In Spain, the general practitioners (GPs) are those mainly in charge of the daily care of older populations. In this regard, Spanish primary care services are mostly used by patients with older age, high risk of morbidity, more complex profile and four or more chronic diseases [56]. Additionally, visits to GPs by older Spanish people increased since 92.4 per cent in 2009 to 94.5 per cent in 2017 [57]. These numbers are relevant in a context where around one-third of professionals at primary care settings experience high levels of burnout [58] and this phenomenon has been exacerbated as a consequence of the COVID-19 pandemic [59]. Thus, according to the good predictive performance of TFI reported in our study, this instrument can be useful as a prognostic support tool for the daily care and early detection of adverse outcomes among older patients in family medicine. This use is aligned with the actions included in the roadmap to approach frailty in Spain through preventive and early detection actions and supporting an integrated and coordinated model of care [60]. This approach could be especially sensitive and efficient in the current pandemic situation where the postponement of regular care and contacts with older people may mean exacerbations of their multiple chronic health problems [61].

Our findings are limited by several issues. On the one hand, a follow-up assessment was performed after 6 months after the baseline assessment. This time can be considered short and could mean a bias when comparing our results with those obtained by other authors using longer time frames, such as a one-year period [28,48,50]. On the other hand, although the sample size was adequate for carrying out statistical analysis of TFI, the sample belonged to a population from only one Spanish region.

## 5. Conclusions

To our knowledge, this is the first study that evaluates the predictive performance of the Spanish version of TFI. This study provides new evidence confirming that the TFI is a valid tool to predict several adverse outcomes in Spanish community-dwelling older adults. Health and care professionals can use this rapid and simple questionnaire to identify levels of frailty among their patients; but also, TFI has been shown to be a good instrument to predict adverse outcomes, such as dependence on performing ADLs or IADLs, decreased quality of life, problems with medication adherence or higher consumption of healthcare resources. In addition, the TFI scores provide key information that may allow professionals to plan and activate health and social care services and resources to support frail patients’ needs at physical, psychological and social levels. In conclusion, the predictive performance of the TFI may allow the system to be more proactive in approaching older adults’ adverse outcomes around frailty in the present and advanced stages. As stated by Sourial et al. (2013) [62], some characteristics of the older population cannot be altered, but frailty may be.

## Figures and Tables

**Table 1:** Characteristics of the Participants (N = 552).

Participants’ Characteristics	Baseline	Follow-Up
Socio-demographic		
Age, mean (SD)	79.1 (6.3)	79.2 (6.2)
Sex, N (%)		
Female	344 (62.3%)	
Male	208 (37.7%)	
Marital status, N (%)		
Married or cohabiting	304 (55.1%)	
Widowed	203 (36.8%)	
Single	26 (4.7%)	
Divorced	19 (3.4%)	
Living situation, N (%)		
Living with others	406 (73.6%)	
Living alone	146 (26.4%)	
Educational level, N (%)		
Primary or less	176 (31.9%)	
Secondary	215 (38.9%)	
Tertiary or higher	160 (29.0%)	
Frailty (TFI)		
TFI Total, mean (SD)	5.6 (3.1)	5.4 (3.0)
TFI Physical, mean (SD)	3.3 (2.0)	3.4 (2.0)
TFI Psychological, mean (SD)	1.3 (1.0)	1.3 (1.0)
TFI Social, mean (SD)	0.9 (0.8)	0.9 (0.8)
Adverse outcomes		
Disability (GARS), mean (SD)	30.1 (16.9)	30.7 (17.3)
Fall risk		
Falls (nº), mean (SD)	0.7 (1.3)	0.7 (1.3)
Fear of falling, N (%) of yes	313 (56.7%)	336 (60.9%)
Falls Efficacy (FES-I), mean (SD)	11.4 (5.9)	11.5 (6.2)
Quality of life		
Physical health (SF-12), mean (SD)	57.4 (30.1)	55.3 (31.5)
Mental health (SF-12), mean (SD)	68.6 (25.7)	66.0 (25.9)
Loneliness (De Jong Gierveld Scale), mean (SD)	1.3 (1.5)	1.2 (1.5)
Use healthcare resources		
Visits GP (nº), mean (SD)	7.9 (7.1)	7.7 (8.5)
Hospital admissions (nº), mean (SD)	0.4 (0.9)	0.6 (1.9)
Polypharmacy, N (%) of yes	275 (49.8%)	271 (49.1%)
Difficulties in taking medications, N (%) of yes	84 (15.2%)	93 (6.8%)

**Table 2:** Correlations between frailty and adverse outcomes.

	Disability	Physical Health	Mental Health	Loneliness	Falls Efficacy	Falls (nº)	Visits GP (nº)	Hospital Admissions (nº)
Baseline								
Total frailty	0.607 **	0.706 **	0.680 **	0.356 **	0.590 **	0.324 **	0.167 **	0.256 **
Physical frailty	0.595 **	0.704 **	0.593 **	0.202 **	0.580 **	0.320 **	0.192 **	0.228 **
Psychological frailty	0.408 **	0.456 **	0.569 **	0.332 **	0.393 **	0.222 **	0.118 **	0.175 **
Social frailty	0.302 **	0.344 **	0.382 **	0.421 **	0.295 **	0.152 **	0.008 (ns)	0.173 **
Follow-up								
Total frailty	0.637 **	0.694 **	0.703 **	0.346 **	0.612 **	0.308 **	0.188 **	0.104 *
Physical frailty	0.579 **	0.693 **	0.611 **	0.208 **	0.569 **	0.282 **	0.223 **	0.97 *
Psychological frailty	0.406 **	0.448 **	0.564 **	0.332 **	0.391 **	0.196 **	0.119 **	0.580 (ns)
Social frailty	0.312 **	0.320 **	0.385 **	0.431 **	0.309 **	0.157 **	0.540 (ns)	0.550 (ns)

Note: ** *p* = 0.01; * *p* = 0.05; ns, not significant.

**Table 3:** CROSS-SECTIONAL Effects of Socio-demographic Characteristics and Frailty Domains on Adverse Outcomes: Linear Regression Analyses.

	Disability			Physical Health			Mental Health			Falls Efficacy			Loneliness			Visits GP			Hospital Admissions			Number of Falls		
	B	SE	*p*	B	SE	*p*	B	SE	*p*	B	SE	*p*	B	SE	*p*	B	SE	*p*	B	SE	*p*	B	SE	*p*
Age	1.073	0.105	0.000	−1.641	0.201	0.000	−1.149	0.173	0.000	0.323	0.040	0.000	−0.018	0.011	0.098	0.052	0.064	0.417	0.031	0.015	0.041	0.043	0.009	0.000
Marital status	1.186	0.424	0.005	−2.190	0.813	0.007	−1.013	0.699	0.148	0.452	0.161	0.005	0.134	0.045	0.003	−0.005	0.256	0.986	−0.038	0.060	0.526	0.000	0.036	0.997
Educational level	−4.017	0.822	0.000	8.866	1.576	0.000	6.206	1.357	0.000	−1.641	0.313	0.000	−0.131	0.086	0.126	−0.027	0.491	0.956	−0.110	0.113	0.333	−0.187	0.070	0.008
Household composition	2.054	0.463	0.000	−1.028	0.892	0.249	−0.767	0.766	0.318	0.218	0.176	0.216	−0.096	0.049	0.050	−0.026	0.282	0.926	0.037	0.066	0.579	0.007	0.039	0.850
ΔR^2^	ΔR^2^ = 0.305; *p* = 0.000			ΔR^2^ = 0.230; *p* = 0.000			ΔR^2^ = 0.159; *p* = 0.000			ΔR^2^ = 0.224; *p* = 0.000			ΔR^2^ = 0.029; *p* = 0.003			ΔR^2^ = 0.029; *p* = 0.003			ΔR^2^ = 0.029; *p* = 0.003			ΔR^2^ = 0.069; *p* = 0.000		
Physical frailty	4.020	0.323	0.000	−9.205	0.519	0.000	−5.367	0.433	0.000	1.426	0.117	0.000	0.005	0.031	0.866	0.893	0.200	0.000	0.081	0.048	0.092	0.143	0.029	0.000
Psychological frailty	2.669	0.627	0.000	−4.813	1.005	0.000	−8.182	0.840	0.000	0.880	0.228	0.000	0.301	0.061	0.000	0.267	0.387	0.490	0.021	0.092	0.821	0.090	0.055	0.105
Social frailty	2.380	0.737	0.001	−3.263	1.182	0.006	−4.496	0.988	0.000	0.861	0.268	0.001	0.633	0.071	0.000	−0.169	0.453	0.709	0.066	0.106	0.534	0.094	0.065	0.149
ΔR^2^	ΔR^2^ = 0.378; *p* = 0.001			ΔR^2^ = 0.514; *p* = 0.001			ΔR^2^ = 0.504; *p* = 0.000			ΔR^2^ = 0.362; *p* = 0.000			ΔR^2^ = 0.227; *p* = 0.000			ΔR^2^ = 0.001; *p* = 0.959			ΔR^2^ = 0.015; *p* = 0.123			ΔR^2^ = 0.090; *p* = 0.000		
R^2^ total	R^2^ = 0.469; F = 68.523; *p* = 0.000			R^2^ = 0.536; F = 89.301; *p* = 0.000			R^2^ = 0.508; F = 79.730; *p* = 0.000			R^2^ = 0.407; F = 53.080; *p* = 0.000			R^2^ = 0.262; F = 27.368; *p* = 0.000			R^2^ = 0.034; F = 2.565; *p* = 0.013			R^2^ = 0.019; F = 1.337; *p* = 0.230			R^2^= 0.110; F = 9.527; *p* = 0.000		

Abbreviations: B, unstandardized coefficients; SE, the standard error of indirect effects estimated.

**Table 4:** CROSS-SECTIONAL Effects of Socio-demographic Characteristics and Frailty Domains on Adverse Outcomes: Logistic Regression Analyses.

	Falls				Fear of Falling				Polypharmacy				Difficulty in Taking Medications			
	B	SE	*p*	OR (95% CI)	B	SE	*p*	OR (95% CI)	B	SE	*p*	OR (95% CI)	B	SE	*p*	OR (95% CI)
Age	0.063	0.015	0.000	1.033–1.097	0.051	0.015	0.001	1.021–1.084	0.104	0.016	0.000	1.075–1.146	0.100	0.019	0.000	1.064–1.148
Marital status (single)	0.205	0.227	0.366	0.787–1.915	0.208	0.229	0.365	0.785–1.930	−0.056	0.230	0.807	0.602–1.485	−0.208	0.290	0.473	0.460–1.435
Educational level (primary studies)	0.534	0.193	0.006	1.168–2.491	0.765	0.206	0.000	1.436–3.215	0.550	0.196	0.005	1.179–2.546	0.547	0.242	0.024	1.074–2.777
Household composition (single)	−0.039	0.249	0.875	0.590–1.567	0.251	0.259	0.333	0.773–2.134	−0.078	0.253	0.758	0.564–1.518	0.035	0.322	0.913	0.551–1.945
χ^2^	χ^2^ = 32.926; *p* = 0.000				χ^2^ = 36.958; *p* = 0.000				χ^2^ = 60.784; *p* = 0.000				χ^2^ = 35.265; *p* = 0.000			
Physical frailty	0.196	0.051	0.000	1.101–1.345	0.436	0.057	0.000	1.382–1.731	0.407	0.055	0.000	1.349–1.672	0.247	0.067	0.000	1.122–1.460
Psychological frailty	0.154	0.097	0.113	0.964–1.411	0.023	0.102	0.822	0.838–1.250	0.167	0.099	0.091	0.973–1.436	0.356	0.126	0.005	1.114–1.829
Social frailty	0.260	0.114	0.022	1.038–1.620	0.245	0.123	0.045	1.005–1.625	0.118	0.117	0.317	0.894–1.416	0.335	0.143	0.019	1.056–1.851
χ^2^	χ^2^ = 43.715; *p* = 0.000				χ^2^ = 101.826; *p* = 0.000				χ^2^ = 103.207; *p* = 0.000				χ^2^ = 52.793; *p* = 0.000			
χ^2^ total	χ^2^ = 54.760; *p* = 0.000				χ^2^ = 110.694; *p* = 0.000				χ^2^ = 118.845; *p* = 0.000				χ^2^ = 65.719; *p* = 0.000			

Abbreviations: B, unstandardized coefficients; SE, the standard error of indirect effects estimated.

**Table 5:** LONGITUDINAL Effects of Socio-demographic Characteristics and Frailty Domains on Adverse Outcomes: Linear Regression Analyses.

	Disability			Physical Health			Mental Health			Falls Efficacy			Loneliness			Visits GP			Hospital Admissions			Number of Falls		
	B	SE	*p*	B	SE	*p*	B	SE	*p*	B	SE	*p*	B	SE	*p*	B	SE	*p*	B	SE	*p*	B	SE	*p*
Age	1.057	0.105	0.000	−1.593	0.201	0.000	−1.139	0.173	0.000	0.318	0.040	0.000	−0.013	0.011	0.235	0.048	0.065	0.460	0.032	0.015	0.035	0.043	0.009	0.000
Marital status	1.363	0.427	0.000	−2.650	0.819	0.001	−1.129	0.705	0.110	0.502	0.162	0.002	0.122	0.044	0.006	0.008	0.259	0.976	−0.039	0.060	0.513	0.003	0.036	0.927
Educational level	−3.984	0.818	0.000	8.835	1.571	0.000	6.162	1.354	0.000	−1.636	0.312	0.000	−0.130	0.085	0.128	−0.29	0.490	0.952	−0.104	0.113	0.357	−0.184	0.07	0.009
Household composition	2.070	0.464	0.000	−1.012	0.893	0.257	−0.806	0.768	0.295	0.221	0.176	0.212	−0.038	0.048	0.427	−0.27	0.283	0.924	0.041	0.067	0.542	0.006	0.039	0.871
ΔR^2^	ΔR^2^ = 0.308; *p* = 0.000			ΔR^2^ = 0.233; *p* = 0.000			ΔR^2^ = 0.159; *p* = 0.000			ΔR^2^ = 0.002; *p* = 0.212			ΔR^2^ = 0.001; *p* = 0.427			ΔR^2^ = 0; *p* = 0.924			ΔR^2^ = 0.001; *p* = 0.542			ΔR^2^ = 0.069; *p* = 0.000		
Physical frailty	4.231	0.345	0.000	−8.062	0.609	0.000	−4.908	0.507	0.000	1.424	0.127	0.000	0	0.034	0.000	0.813	0.211	0.000	0.120	0.50	0.016	0.144	0.030	0.000
Psychological frailty	1.891	0.679	0.006	−4.316	1.199	0.000	−7.148	0.999	0.000	0.553	0.251	0.028	0.249	0.068	0.000	−0.409	0.415	0.326	−0.031	0.098	0.749	0.078	0.059	0.185
Social frailty	1.819	0.765	0.018	−2.624	1.349	0.000	−3.113	0.0126	0.006	0.741	0.283	0.009	0.505	0.076	0.000	0.052	0.464	0.910	−0.024	0.108	0.824	0.064	0.066	0.338
ΔR^2^	ΔR^2^ = 0.349; *p* = 0.001			ΔR^2^ = 0.386; *p* = 0.000			ΔR^2^ = 0.374; *p* = 0.000			ΔR^2^ = 0.309; *p* = 0.000			ΔR^2^ = 0.143; *p* = 0.000			ΔR^2^ = 0.031; *p* = 0.001			ΔR^2^ = 0.013; *p* = 0.087			ΔR^2^ = 0.08; *p* = 0.000		
R^2^ total	R^2^ = 0.452; F = 63.877; *p* = 0.000			R^2^ = 428; F = 57.746; *p* = 0.000			R^2^ = 0.392; F = 49.840; *p* = 0.000			R^2^ = 0.366; F = 44.64; *p* = 0.000			R^2^ = 0.165; F = 12.294; *p*= 0.000			R^2^ = 0.034; F = 2.565; *p*= 0.013			R^2^ = 0.021; F = 1.490; *p* = 0.169			R^2^ = 0.103; F= 8.937; *p* = 0.000		

Abbreviations: B, unstandardized coefficients; SE, the standard error of indirect effects estimated.

**Table 6:** LONGITUDINAL Effects of Socio-demographic Characteristics and Frailty Domains on Adverse Outcomes: Logistic Regression Analyses.

	Falls				Fear of Falling				Polypharmacy				Difficulty in Taking Medications			
	B	SE	*p*	OR (95% CI)	B	SE	*p*	OR (95% CI)	B	SE	*p*	OR (95% CI)	B	SE	*p*	OR (95% CI)
Age	0.063	0.015	0.000	1.065 (1.033–1.097)	0.047	0.015	0.002	1.048 (1.017–1.080)	0.105	0.016	0.000	1.111 (1.07–1.147)	0.099	0.019	0.000	1.104 (1.063–1.147)
Marital status (unmarried, widow/er)	0.264	0.231	0.253	1.302 (0.828–2.050)	0.398	0.238	0.094	1.488 (0.934–2.371)	0.038	0.236	0.873	1.038 (0.653–1.650)	−0.092	0.293	0.752	0.912 (0.514–1.618)
Educational level (primary education)	0.527	0.193	0.006	1.693 (1.159–2.473)	0.758	0.206	0.000	2.134 (1.425–3.194)	0.525	0.197	0.008	1.691 (1.150–2.486)	0.523	0.242	0.030	1.688 (1.051–2.712)
Household composition (living alone)	−0.093	0.255	0.714	0.911 (0.553–1.501)	0.122	0.267	0.649	1.129 (0.669–1.906)	−0.188	0.260	0.468	0.828 (0.498–1.378)	−0.007	0.325	0.983	0.993 (0.526–1.876)
χ^2^	χ^2^ = 33.880; *p* = 0.000				χ^2^ = 38.742; *p* = 0.000				χ^2^ = 62.711; *p* = 0.000				χ^2^ = 35.427; *p* = 0.000			
Physical frailty	0.175	0.053	0.001	1.191 (1.075–1.321)	0.392	0.58	0.000	1.481 (1.321–1.659)	0.348	0.055	0.000	1.416 (1.271–1.578)	0.334	0.072	0.000	1.397 (1.212–1.610)
Psychological frailty	0.209	0.102	0.041	1.232 (1.008–1.506)	0.133	0.108	0.220	1.142 (0.924–1.411)	0.176	0.104	0.090	1.193 (0.973–1.462)	0.360	0.134	0.007	1.434 (1.103–1.863)
Social frailty	0.204	0.115	0.075	1.227 (0.980–1.536)	0.107	0.122	0.380	1.113 (0.876–1.415)	0.223	0.118	0.059	1.249 (0.992–1.573)	0.232	0.146	0.112	1.261 (0.948–1.679)
χ^2^	χ^2^ = 39.734; *p*= 0.000				χ^2^ = 88.247; *p* = 0.000				χ^2^ = 91.140; *p* = 0.000				χ^2^ = 61.669; *p* = 0.000			
χ^2^ total	χ^2^ = 52.447; *p* = 0.000				χ^2^ = 97.845; *p* = 0.000				χ^2^ = 113.927; *p* = 0.000				χ^2^ = 72.651; *p* = 0.000			

Abbreviations: B, unstandardized coefficients; SE, the standard error of indirect effects estimated.

## Data Availability

Not applicable.

## Supplementary Materials

The following supporting information can be downloaded at: https://www.mdpi.com/article/10.3390/ijerph191912756/s1.
