# Supplementary material for: Frailty as a Predictor of Adverse Outcomes among Spanish Community-Dwelling Older Adults
Source: Int J Environ Res Public Health. 2022 Oct 5;19(19):12756. doi: 10.3390/ijerph191912756 (PMC9566344; doi:10.3390/ijerph191912756)
Supplement: Supplementary file 1 [file ijerph-19-12756-s001.zip › ijerph-1872884-supplementary.pdf]

Table S1. Receiver operating curve (ROC) OUTCOMES for TFI with respect to the adverse outcomes

Cut-off Point	Adverse outcome	Cross-sectional			Longitudinal		
		Sensitivity	Specificity	AUC (95% CI)	Sensitivity	Specificity	AUC (95% CI)
≥ 5	Disability	0.971	0.617	0.875 (0.846 - 0.905)	0.951	0.584	0.844 (0.810 - 0.878)
≥ 6		0.903	0.733		0.893	0.699	
≥ 5	Physical health	0.876	0.772	0.875 (0.846 - 0.905)	0.832	0.701	0.822 (0.787 - 0.857)
≥ 6		0.735	0.855		0.717	0.799	
≥ 5	Mental health	0.923	0.657	0.869 (0.837 - 0.901)	0.852	0.600	0.809 (0.767 - 0.851)
≥ 6		0.817	0.765		0.782	0.718	
≥ 5	Loneliness	0.781	0.527	0.694 (0.649 - 0.739)	0.684	0.571	0.692 (0.646 - 0.737)
≥ 6		0.663	0.596		0.588	0.681	
≥ 5	Falls efficacy	0.912	0.661	0.856 (0.823 - 0.889)	0.878	0.616	0.808 (0.769 - 0.848)
≥ 6		0.816	0.772		0.803	0.733	
≥ 5	Falls	0.634	0.591	0.668 (0.622 - 0.715)	0.693	0.588	0.659 (0.613 - 0.706)
≥ 6		0.537	0.703		0.561	0.677	
≥ 5	Fear of falling	0.628	0.718	0.730 (0.688 - 0.773)	0.658	0.704	0.723 (0.680 - 0.766)
≥ 6		0.503	0.838		0.548	0.801	
≥ 5	Polypharmacy	0.674	0.685	0.731 (0.690 - 0.773)	0.696	0.659	0.728 (0.685 - 0.770)
≥ 6		0.549	0.774		0.593	0.767	
≥ 5	Difficulty taking medication	0.777	0.566	0.745 (0.693 - 0.797)	0.798	0.541	0.746 (0.695 - 0.798)
≥ 6		0.691	0.677		0.713	0.651	

Abbreviations: AUC, Area under the curve; CI, confidence interval.
